# Narrative review of the development of an ischaemic heart disease prognostic scoring tool (i-IHD score) among patients with type 2 diabetes mellitus in Malaysia

**DOI:** 10.51866/rv.1027

**Published:** 2026-06-07

**Authors:** Muhammad Muzzammil Mohamad Salleh, Sazzli Shahlan Kasim, Tajul Rosli Razak, Nazar Mohd Azahar, Mohamad Rodi Isa

**Affiliations:** 1 Department of Public Health, Faculty of Medicine, Universiti Teknologi MARA, Sungai Buloh, Selangor, Malaysia.; 2 Cardiovascular Advancement and Research Excellence Institute (CARE Institute), Universiti Teknologi MARA, Sungai Buloh, Selangor, Malaysia.; 3 Faculty of Computer and Mathematical Sciences, Universiti Teknologi MARA, Shah Alam, Selangor, Malaysia.; 4 Department of Medical Laboratory Technology, Faculty of Health Sciences, Universiti Teknologi MARA (UiTM) Cawangan Pulau Pinang, Kampus Bertam, Kepala Batas, Pulau Pinang, Malaysia.

**Keywords:** Myocardial ischaemia, Diabetes mellitus, Type 2, Artificial intelligence, Prognosis, Malaysia

## Abstract

**Introduction:**

Ischaemic heart disease (IHD) remains a major cause of mortality among individuals with type 2 diabetes mellitus (T2DM) in Malaysia. Conventional cardiovascular risk models, such as the Framingham risk score, often show limited calibration in Asian populations. Artificial intelligence (Al)-calibrated models have emerged as potential alternatives, yet their generalisability and clinical utility across different populations remain uncertain. This narrative review aimed to summarise existing prognostic models for IHD in patients with T2DM and identify methodological gaps relevant to the development of a locally calibrated model.

**Methods:**

This narrative review employed a structured search strategy guided by PRISMA principles but was not conducted as a full systematic review. We synthesised evidence from epidemiological and prognostic research. Studies comparing conventional statistical approaches (e.g. logistic regression and Cox models) with AI-calibrated models such as extreme gradient boosting, random forest and support vector machines were reviewed.

**Results:**

Eleven studies met the inclusion criteria; four used conventional statistical methods, and seven applied AI or machine learning algorithms. The reported discrimination (area under the curve=0.66–0.94) varied widely. Conventional models commonly lacked external validation and demonstrated restricted applicability beyond their original cohorts. AI-calibrated models showed promising discrimination in some datasets but similarly experienced limited validation and lacked benchmarking against traditional statistical methods. Across the studies, limited calibration and validation reduced generalisability to heterogeneous Malaysian populations.

**Conclusion:**

Developing a locally AI-calibrated i-IHD score could enable early risk identification, guide targeted interventions and support national health initiatives, including the Health White Paper 2023 and 13th Malaysia Plan.

## Introduction

Ischaemic heart disease (IHD) remains one of the leading contributors to the global burden of cardiovascular disease.^1^ Among patients with diabetes mellitus, the burden is particularly substantial, with 5.11% reported to have IHD complications according to the National Diabetes Registry (NDR).^2^ Furthermore, IHD has been identified as a leading cause of mortality among individuals with diabetes.^3^ In Malaysia, regional disparities are evident as the NDR 2023 report indicates that Johor has a 4.3% larger proportion of patients with both diabetes and IHD than the entire nation, highlighting a substantial local burden.^2^

Evidence from previous studies further underscores the importance of prognostic research in assessing IHD risk among patients with type 2 diabetes mellitus (T2DM). The prospective cohort study by Huang et al.^4^ involving 1021 individuals with a mean age of 67.9 years reported that 25.0% developed cardiovascular disease over a median follow-up of 10.6 years. Similarly, Lee et al.,^5^ using a Cox proportional hazards model, identified 24,809 cases of myocardial infarction (1.9%) over a 7-year follow-up period. In a retrospective cohort study employing machine learning methods, Sang et al.^6^ reported a prevalence of 10.2% for coronary heart disease. Additionally, the logistic regression-based prognostic study by Shi et al.^7^ found that 16.46% of patients with T2DM developed IHD, with a mean age of 65 years. The sex-specific analyses by Wan et al.^8^ demonstrated that the 5- and 10-year cardiovascular event rates were higher in men (22.0% and 23.83%, respectively) than in women (18.63% and 20.43%, respectively). Moreover, this study conducted in China and externally validated in Scotland reported cardiovascular disease incidence rates of 22.25% and 7.31%, respectively, over a median follow-up of 4.75 years.

Collectively, the abovementioned findings highlight the urgent need to address the dual burden of diabetes mellitus and IHD. The complex interplay between these conditions necessitates the development of accurate and reliable prognostic tools to facilitate early risk identification and timely intervention among high-risk patients. The wide variation in reported IHD prevalence, ranging from 1.9% to 25.0%, reflects heterogeneity in population characteristics, study design and analytical approaches. The complexity of predicting cardiovascular outcomes among patients with T2DM is underlined by the disparities and diverse methodologies used, including Cox regression, logistic regression and machine learning models.

### Situational analysis of IHD in Malaysia

IHD is the leading cause of death in Malaysia.^9^ According to the National Health Morbidity Survey 2023, the increasing prevalence of diabetes, especially T2DM, in the state is strongly associated with the rising incidence of IHD.^10^

Currently, Malaysia implements several strategies to cope with the rising burden of IHD among patients with diabetes. Based on the National Strategic Plan for Non-Communicable Disease (NSP-NCD), health initiatives are guided by this strategic framework through multi-sectoral action such as *Komuniti Sihat Perkasa Negara*, which promotes healthy lifestyles in the community through local volunteerism. This initiative requires the involvement of multiple agencies, including local municipal and community organisations. Other initiatives under the NSP-NCD include health promotion and the strengthening of community-based screening, such as the PEKA B40 health screening programme, with a focus on early detection and prompt intervention.

Existing evidence emphasises the urgent need for targeted and context-specific interventions to mitigate the growing burden of IHD-related morbidity and mortality among patients with diabetes in Malaysia. This highlights the critical role of early risk stratification and timely clinical management in preventing adverse cardiovascular outcomes. Consequently, the development of a locally adapted prognostic model, such as the i-Heart score, is warranted to improve risk prediction in this population. The application of rigorous internal and external validation, alongside artificial intelligence (AI) calibration across multiple phases, is expected to enhance the predictive performance, validity and generalisability of the model.

### Prognostic scoring tool for IHD among patients with diabetes

Research studies have different types, one of which is prognostic research, which involves predicting the likelihood of future health outcomes by analysing individual characteristics and relevant predictors. This type of research uses multivariable models to identify significant predictors and estimate the probabilities of specific outcomes such as disease progression, recovery or mortality. Often referred to as a prognostic tool or risk score, a predictive model plays an important role in enhancing clinical decision-making and patient outcomes across various medical disciplines.^11^

In Malaysia, the Framingham risk score (FRS) has been widely utilised as a tool for predicting the risk of IHD in various populations, including patients with T2DM receiving treatment in government primary care facilities in Malaysia. However, its application among patients with diabetes in Malaysia presents several challenges and limitations that may affect its predictive accuracy and clinical utility.

Studies have indicated that the FRS tends to underestimate the risk of cardiovascular events in populations with a high prevalence of diabetes. For instance, Metcalf et al.^12^ found that the FRS underestimates the 10-year coronary heart disease risk in individuals with T2DM. This underestimation can lead to inadequate preventive measures and treatment strategies for patients with diabetes in Malaysia, who are already at a heightened risk for IHD due to the interplay of diabetes and other cardiovascular risk factors such as hypertension and dyslipidaemia. Furthermore, the FRS does not incorporate the effects of diabetes duration and control, which are critical in assessing cardiovascular risk among patients with diabetes. Research has shown that a longer duration of diabetes and poor glycaemic control significantly increase the risk of IHD.^13^ The failure to include these variables in the FRS may limit its effectiveness in predicting IHD among Malaysian patients with diabetes, who often present with varying degrees of diabetes management.

Another substantial issue with the FRS is its original development in a predominantly Caucasian population, which may not accurately reflect the risk profiles of Malaysian populations, particularly those with diabetes. The FRS was derived from data that primarily included individuals with lifestyles, dietary habits and genetic backgrounds different from the Malaysian demographic.^14^ This lack of generalisability raises concerns about the applicability of the FRS in predicting IHD among Malaysian patients with diabetes, who may exhibit different risk factor distributions and cardiovascular disease patterns.

Studies have also shown that patients with diabetes often exhibit higher levels of fasting blood sugar and glycated haemoglobin (HbA1c) which are associated with an increased risk of developing IHD. The findings of Elias and Al-Shammaa^15^ support this, indicating that elevated insulin resistance and glucose levels are significant predictors of IHD in populations with diabetes. As the Malaysian population continues to experience rising rates of obesity and sedentary lifestyles, the prevalence of insulin resistance is likely to increase, further heightening the risk of IHD among patients with diabetes.

Moreover, a rigorous process is required in prognostic studies, including model development and validation, to provide evidence-based predictions.^16^ The integration of diverse significant predictors is also pivotal for refining models, as highlighted by Peeperkorn et al.^17^ The authors also emphasised that such integration broadens the applicability of prognostic models across different healthcare settings and enhances predictive accuracy through a multifaceted approach.

Several frameworks and checklists can be used to appraise prognostic studies, one of which is the TRIPOD-AI statement. The Transparent Reporting of a Multivariable Prediction Model for Individual Prognosis or Diagnosis guideline was recently updated in 2024 to incorporate AI-specific elements. With the growing integration of AI in prognostic models, the statement has expanded its checklist from 22 to 27 items. Overall, the guideline aims to promote holistic, accurate and transparent reporting of prognostic studies, particularly in model development and performance evaluation.^18^

As prognostic studies have become increasingly prevalent in the medical literature, the need for structured guidance led to the development of the Critical Appraisal and Data Extraction for Systematic Reviews of Prediction Modelling Studies tool, which provides researchers with a systematic approach to appraising prediction models.^19^

### Concepts of prognostic research

**Figure 1:**
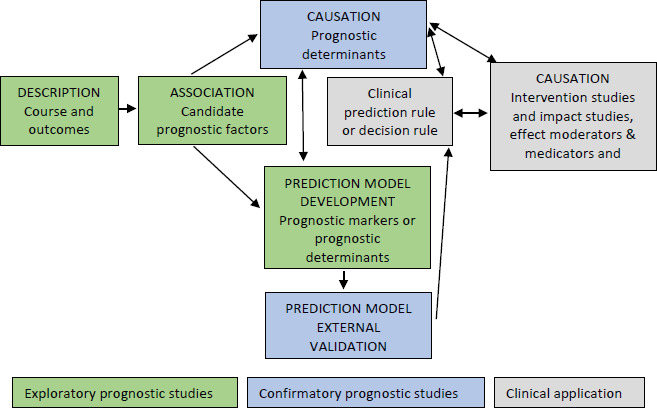
Conceptual framework for prognostic research.

In their study, Kent et al.^20^ proposed four main objectives of prognostic research: description, association, prediction and causation. These objectives were further classified into two types of studies, namely exploratory and confirmatory studies. Exploratory studies involve description, association and development of prediction models, whereas confirmatory studies focus on the external validation of prognostic models and the investigation of causalrelationships. Aconceptual diagram is presented in Figure 1 to clarify rhis framework.

In the proposed concept, three major steps are included: the exploratory step, which envolves description, associafion, development of prognostic models and internal validation of such models; the confirmatory step, which includes assessing the generalisability and reliability of models in independent populations; and the clinical application step, which translates models into clinical prediction rules for practical use.

## Methods

This narrative review synthesised evidence on prognostic tools for IHD among patients with T2DM, with particular emphasis on models incorporating AI calibration. A structured and comprehensive literature search was conducted across four electronic databases (PubMed, Scopus, Web of Science and Google Scholar) for studies published from January 2015 to June 2025. No trial or study registers (e.g., ClinicalTrials.gov or PROSPERO) were included, as the objective was to review published prognostic model studies rather than ongoing trials.

**Figure 2:**
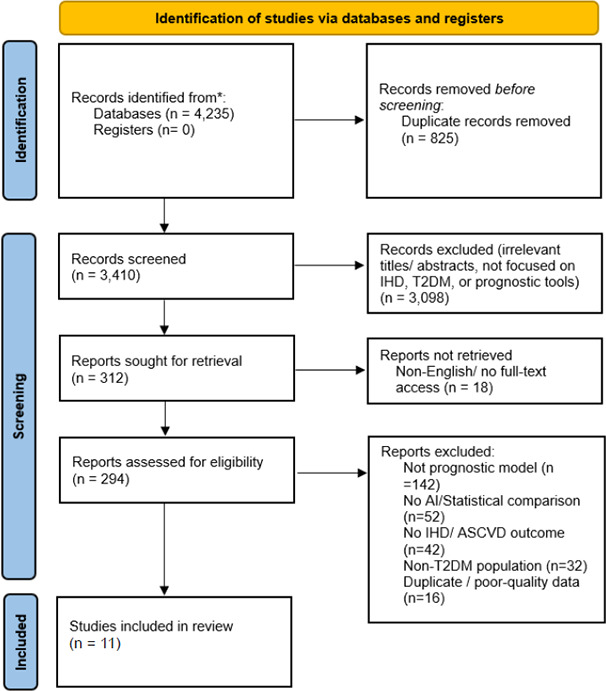
Flow diagram of study selection for the review of AI-calibrated prognostic models for IHD among patients with T2DM. IHD: ischaemic heart disease; T2DM: type 2 diabetes mellitus; AI: artificial intelligent; ASCVD: atherosclerotic cardiovascular disease.

The search strategy combined controlled vocabulary and free-text terms including ‘ischemic heart disease’, ‘type 2 diabetes mellitus’, ‘prognostic model’, ‘risk score’, ‘prediction model’ and ‘artificial intelligence’. Reference lists of relevant articles were also screened to identify additional studies. A structured search was conducted, and records were screened; the selection process is summarised in Figure 2.

Studies were eligible when they (1) involved adult populations with T2DM; (2) developed or validated prognostic or predictive models for IHD or atherosclerotic cardiovascular disease; and (3) applied either conventional statistical methods (e.g., logistic regression and Cox models) or AI-calibrated algorithms (e.g., random forest, extreme gradient boosting [XGBoost] and support vector machines).

Studies were excluded when they were not published in English, lacked full-text availability, involved populations without diabetes or did not present model performance metrics. Data extraction focused on study design, population characteristics, prognostic variables, model type, validation approach and performance indicators. Several evaluation metrics were used to evaluate the performance of the prognostic model, including the receiver operating characteristic (ROC) curve, generating the area under the curve (AUC) as well as specificity and sensitivity. An AUC of 1 indicated perfect discriminatory ability, while an AUC of 0.5 indicated no discriminatory power.^21^

This narrative review employed a structured search strategy guided by PRISMA principles but was not conducted as a full systematic review. The findings were summarised narratively, highlighting methodological aspects, validation practices and evidence gaps relevant to the development of a locally AI-calibrated prognostic scoring tool (i-IHD score) for Malaysian populations with diabetes. Figure 2 illustrates the data collection flowchart.

## Results

### Available prognostic models for IHD among patients with T2DM

The initial database search identified a total of 4235 records. After the removal of 825 duplicate entries, 3410 unique records remained for title and abstract screening. Of them, 3098 were excluded, as they did not meet the inclusion criteria. A total of 312 full-text articles were retrieved for detailed assessment; 18 could not be accessed in full or were non-English publications. The remaining 294 articles were evaluated for eligibility, from which 283 were excluded due to reasons as shown in Figure 2. Ultimately, 11 studies met the inclusion criteria and were included in this narrative review; four used conventional statistical approaches without AI integration, and seven incorporated AI or machine learning algorithms. Table 1 and Table 2 summarise the main characteristics of these studies.

**Table 1:** Summary of prognostic models for IHD among patients with T2DM without artificial intelligence integration.

Author, year	Country	Study design/population	Finding	Limitation
Qin et al., 2020^22^	China	Prospective cohort of patients with T2DM post-PCI (n=2356)	The atherogenic index of plasma predictive model achieved an AUC of 0.82–0.90 for prognostic prediction	No external validation in an independent cohort
Hippisley-Cox and Coupland, 2015^23^	United Kingdom	Retrospective cohort of patients with diabetes from the QResearch primary care database (n=772,739)	Reported AUC of 0.75–0.85 for heart disease prediction among patients with diabetes	No external validation
Koshkina et al., 2022^24^	Russia	Retrospective study among adults with T2DM and comorbid IHD (n=126)	Developed prognostic model demonstrating an AUC of 0.85–0.90, showing good discriminatory performance	Lacked external validation
Xiang et al., 2021^25^	China	Retrospective cohort of patients with both diabetes and coronary heart disease (n=95)	Reported AUC of 0.99 for the combined biomarker model, indicating substantially high internal discrimination	No independent validation

**Table 2:** Evidence on AI-calibrated models for prognostic scoring of IHD among patients with T2DM.

Author (year)	Country	Study design/sample size	Performance matrix	Validation	Calibration of AI with statistical details for each phase
Kee et al. (2023)^30^	Malaysia	Global systematic review (10 studies on diabetes complications)	The highest-performing model (ANN) achieved an AUC of 0.91, a sensitivity of 88.06%, a precision of 76.6% and an accuracy of 87.5%.	Not applicable	None reported
Chu et al. (2021)^31^	China	Cross-sectional ML analysis of adults with T2DM from the MIMIC-III hospital database (n=834)	The ANN demonstrated a sensitivity of 87.5%-88.06%, an accuracy of 87.5% and an AUC of 0.91; these values were derived from ensemble ANN models predicting cardiovascular disease risk among patients with T2DM.	Internal validation only	None reported
Xu et al. (2022)^32^	China	Retrospective cohort study among elderly patients with diabetes mellitus (DM) (Development, n = 23,167; Validation, n = 7,447)	XGBoost demonstrated the best performance, achieving an AUC of 0.851 (95% CI: 0.841-0.861) in the testing dataset and 0.880 (95% CI: 0.872-0.887) in the independent validation dataset.	Internal and external validation	None reported
Lee et al. (2024)^33^	Korea	Retrospective cohort study of newly diagnosed T2DM (n=5040)	AUROC of 0.830 (95% CI=0.818-0.842) in the derivation cohort and 0.722 (95% CI=0.660-0.783) in the validation cohort	Internal and external validation	None reported
Sang et al. (2024)^34^	South Korea	Retrospective study of two independent Korean dataset cohorts (discovery, n=12,809; validation, n=2019)	RF showed the highest AUROC of 0.830 (95% CI=0.818-0.842) in the discovery cohort and 0.722 (95% CI=0.660-0.783) in the validation cohort.	Internal and external validation	None reported
Chen et al. (2025)^35^	China	Retrospective multicentre study of 2517 patients with T2DM who underwent coronary angiography (1943 with CHD; 574 without CHD)	XGBoost (RFE + LightGBM) achieved the best performance: AUC of 0.94 (95% CI of 0.91-0.96), accuracy of 0.902, precision of 0.889, sensitivity of 0.905 and specificity of 0.891.	Internal and external validation	None reported
Abas et al. (2025)^36^	Malaysia	Retrospective cohort study using National Diabetes Registry data from southern Malaysian public clinics (2011–2021); 90,933 patients with T2DM	The LightGBM model achieved an AUC of 0.66 for IHD prediction; the other algorithms (RF, XGBoost and ANN) showed an AUC of 0.58-0.65.	Internal validation	None reported

ANN: artificial neural network, IHD: ischaemic heart disease, RF: random forest, LightGBM: light gradient boosting machine.

Numerous recent prognostic studies on IHD among patients with diabetes were conducted without the integration of AI. In their prospective study, Qin et al.^22^ reported ROC values ranging from 0.82 to 0.90. Conversely, the retrospective cohort studies by Hippisley-Cox and Coupland,^23^ Koshkina et al.^24^ and Xiang et al.^25^ reported AUCs ranging from 0.75 to 0.99. Information regarding external validation in independent cohorts or populations was not reported in these studies. Table 1 presents a summary of the previous literature on prognostic scoring tools for IHD among patients with T2DM without AI calibration.

### AI-calibrated prognostic models for IHD among patients with T2DM

AI has several subsets, including machine learning and deep learning. Machine learning focuses on structured data analysis, such as tabular and numerical values.^26^ Conversely, deep learning is frequently used for unstructured data analysis, including graphics, X-rays, ECGs, videos and images.^27^ Both types of AI have rapidly evolved, enabling algorithmic solutions across diverse domains. Machine learning algorithms such as XGBoost have shown high efficacy in the healthcare domain, particularly in predictive analysis and structured data analysis.^28^ In their study, Pathak et al.^29^ emphasised the contributions of deep learning algorithms such as the recurrent neural network, which excels in handling unstructured data and extracting complex features hierarchically. Multiple studies demonstrated strong performance of their algorithms in predictive analysis for IHD among patients with diabetes.

The studies by Kee et al.^30^ and Chu et al.^31^ demonstrated that artificial neural network models exhibited sensitivity values ranging from 87.50% to 88.06%. Another frequently used AI algorithm was XGBoost, which showed area under the receiver operating characteristic curve (AUROC) values ranging from 0.781 to 0.880.^32,33^ In contrast, the random forest model showed an AUROC of 0.830 (95% CI=0.818-0.842).^34^

Table 2 shows a summary of the previous literature on prognostic scoring tools for IHD among patients with T2DM with AI calibration. The evidence was obtained from various databases and involved studies from different regions of the world. A comparative analysis between statistical models and AI-calibrated models across different phases was not reported in the included studies.

## Discussion

### Rationale for developing the IHD prognostic scoring tool (i-IHD score) for patients with T2DM in Malaysia

Despite the rising burden of IHD among individuals with T2DM, there remains limited literature on the development and validation of prognostic scoring tools in Malaysia and other Southeast Asian countries. The included studies originated primarily from China, South Korea and the United Kingdom, reflecting research activity concentrated in East Asian and European contexts.^22,23,25,31,33-35,37^ Most demographic, genetic and healthcare system factors differ substantially from those in Malaysia. Existing cardiovascular risk prediction models applied in Malaysian settings have demonstrated only moderate discrimination^36^ and calibration issues when used among local populations, including cohorts with T2DM, which may reduce the accuracy of risk stratification and constrain early, targeted prevention at the primary care level.^33,38,39^ This underscores the need for calibrating AI to local data.

The majority of the studies included in this review adopted a retrospective cohort design, primarily utilising existing clinical or registry-based datasets.^22-24,33-36,40^ This design has proven valuable in prognostic model development, as it allows access to large, real-world populations and long-term outcome data at relatively low cost.^41^ Several retrospective studies from China and South Korea achieved excellent model discrimination, with AUROC values ranging from 0.78 to 0.94, indicating that retrospective data can yield robust predictive performance when appropriate pre-processing and validation are applied.^33,34^ The retrospective approach also enables exploration of diverse predictors routinely collected in electronic health records, including metabolic, demographic and treatment-related variables that are essential for IHD risk estimation among populations with diabetes.

Most of the reviewed studies demonstrated satisfactory internal model performance; however, external validation was rarely performed,^23,25,30,34-36,40^ limiting the generalisability and clinical applicability of the prognostic tools.^20^ External validation is crucial in evaluating whether a model maintains its predictive accuracy when applied to an independent population that differs from the one used in model development. Without this step, there is a substantial risk of model overfitting, where the prediction performs well in the development dataset but poorly in real-world clinical settings. Several studies from China and South Korea, although reporting high AUC values of 0.78–0.94, did not test their models in populations beyond their original cohort. Consequently, the absence of external validation reduces the reliability of the tools for use among diverse ethnic and clinical profiles, such as those in Malaysia’s multi-ethnic population.^42^

The need for broader validation is also underscored by Hani and Ahmad,^43^ who conducted a systematic review and found that many existing studies did not adequately evaluate the predictive performance of their models across diverse patient populations. This lack of external validation limits the generalisability of the findings and raises questions about the robustness of the AI scoring systems. Moreover, the implications of overfitting are particularly pronounced in clinical settings where misclassification can lead to inappropriate treatment decisions. The research underscores the importance of employing machine learning algorithms that are not only accurate but also validated across various demographics and clinical settings to avoid the pitfalls of overfitting.44 Without proper validation, there is a risk that AI models may mislead clinicians, resulting in either unnecessary interventions or missed opportunities for critical care.

While several international studies reported higher discriminatory performance, often with AUROC values exceeding 0 . 80,^23-25,32-34^ Malaysian studies generally demonstrated more modest AUROC values,^35^ which may reflect reliance on homogeneous datasets that demonstrate reduced discrimination when applied to more heterogeneous populations, such as those encountered in routine clinical practice due to the absence of external validation procedures.^45^ Without validation across independent populations, model performance estimates may be unstable and less generalisable. These findings suggest that the observed differences in predictive performance are likely attributable to methodological and data-related factors rather than inherent limitations of the Malaysian population, underscoring the importance of external validation and recalibration when developing locally applicable prognostic models.^46^

Several methodological limitations were identified across both conventional statistical and AI-based prognostic models. There was substantial heterogeneity in the selection and definition of prognostic variables,^29,30,32^ with inconsistent inclusion of clinically relevant factors such as duration of diabetes and glycaemic control,^23,25^ potentially affecting model robustness and comparability. In addition, the model development strategies varied considerably across the studies, including logistic regression, Cox proportional hazards models and machine learning algorithms. In several studies, the reporting of model development procedures was limited, with insufficient detail on variable selection methods and handling of missing data.^23,25,29,30^ This lack of methodological transparency may increase the risk of bias and reduce reproducibility.^42^ Furthermore, although most studies reported model discrimination using AUCs, calibration assessment was inconsistently reported, despite its importance in evaluating agreement between predicted and observed outcomes.^23,25,29,31,32,35,40^

Therefore, the i-IHD score, an AI-calibrated model, should be developed and validated to enhance the reliability and applicability of models in clinical practice. This approach will not only improve the predictive accuracy of AI models but also foster greater trust among healthcare professionals in utilising these advanced tools for patient care.

### Methodology of the development of the IHD prognostic scoring tool (i-IHD score) with AI calibration

**Figure 3:**
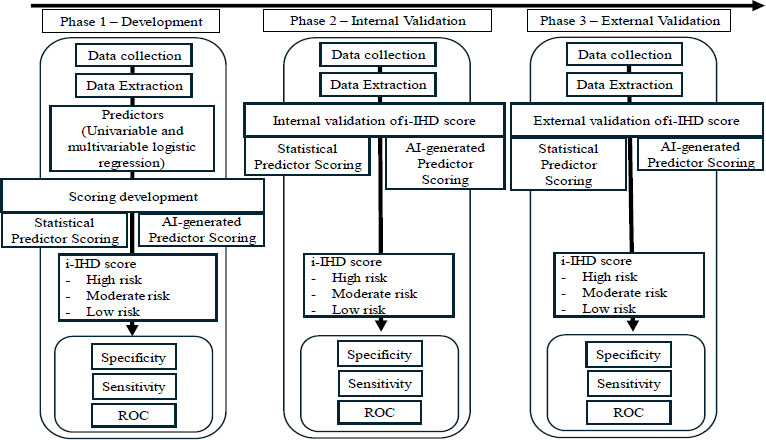
Phaseo oOthe development and validation of the i-IHD score.

The IHD prognostic scoring tool as an AI-calibrated model (i-IHD score) will be developed using a retrospective study based on secondary data from the NDR of Malaysia from 2019 to 2024. The methodology will be divided into three main phases. In Phase 1a, significant predictors of IHD among patients with T2DM will be identified using appropriate statistical analyses. Phase 1b will focus on developing the i-IHD score using conventional statistical methods and then calibrating it with machine learning algorithms to enhance predictive accuracy. In Phase 2, the scoring tool will undergo internal validation using a different cohort of data to assess performance and reproducibility. Finally, Phase 3 will involve external validation in an independent dataset to evaluate thegeneralisability of the tool across different populations. *A* schematic summary of this multi-phase process is illustrated in Figure 3.

The predictive accuracy of the statistical model will be compared with that of the A--calibrated model. Model performance will be evaluated using AUROCs, along with metrics including sensitivity and specificity. 'The goal is to determino whether AI integratian cnn enhanne pre dictive capability compared with conventional statistical methods while minimising overfitting and maximising clinical applicability.

### Clinical significance of the i-IHD score

The development of the AI-calibrated model is relevant, as it will provide valuable insights into key predictors of IHD among patients with T2DM in Malaysia, addressing the limitations of existing tools such as the FRS by incorporating crucial factors such as HbA1c levels. Using realtime data from the NDR as in other studies,^35,47^ the tool will enable early identification and prioritisation of high-risk individuals, improve targeted interventions and optimise healthcare resources. Through rigorous validation, the AI-calibrated model aims to enhance predictive accuracy compared with conventional methods, supporting its integration into public health frameworks in Malaysia in line with the Health White Paper and RMK-13 objectives, ultimately strengthening preventive strategies and reducing the national burden of IHD.

## Conclusion

This review synthesised existing evidence on prognostic models for IHD among patients with T2DM. Conventional models commonly lacked external validation and demonstrated restricted applicability beyond their original cohorts. AI-calibrated models showed promising discrimination in some datasets but similarly experienced limited validation and lacked benchmarking against traditional statistical methods. Moreover, the lack of external validation underscores the need for locally AI-calibrated models, including the i-IHD score, designed to enhance early cardiovascular risk detection and guide preventive management in primary care practice. By leveraging Malaysia’s NDR, this initiative supports precision public health planning and promotes equitable, data-driven care delivery.

## Review protocol registration

This narrative review was not prospectively registered, as it was conducted as a narrative synthesis rather than a systematic review.

## Data sharing statement

The datasets generated and/or analysed during the current study are not publicly available but are available from the corresponding author upon reasonable request.

**How does this paper make a difference in general practice?**

- In general practice, the i-IHD score can support early detection of ischaemic heart disease (IHD) risk among patients with type 2 diabetes mellitus, enabling timely intervention before complications develop.
- This review summarises available evidence on traditional statistical and AI- or machine learning-based prognostic models for IHD risk prediction among patients with type 2 diabetes mellitus, highlighting methodological gaps in model development, calibration and validation.
- Integrating the i-IHD score into routine National Diabetes Registry-based screening aligns with national health priorities, allowing general practitioners to optimise resource allocation and strengthen preventive care strategies in primary care settings.
